# Supplementary material for: A Multicenter Cross-Sectional Survey of Knowledge, Attitude, and Practices of Healthcare Professionals towards Antimicrobial Stewardship in Ghana: Findings and Implications
Source: Antibiotics (Basel). 2023 Sep 29;12(10):1497. doi: 10.3390/antibiotics12101497 (PMC10604649; doi:10.3390/antibiotics12101497)
Supplement: Supplementary file 1 [file antibiotics-12-01497-s001.zip › antibiotics-2614211-supplementary.pdf]

Supplementary Table - TABLE S1 - QUESTIONNAIRE FOR DATA COLLECTION

SECTION A: Socio demographics of Healthcare Professionals (HCPs)

1. Age: ……………………………(years)
2. Sex: Male ☐ Female ☐ Other ☐
3. Name of Hospital: ……………………………………...
4. Level of facility: Primary ☐ Secondary ☐ Tertiary ☐
5. Professional designation?: Medical doctor ☐ Pharmacist ☐ Nurse ☐
6. If you are a medical doctor, what is your specialty?

   Medicine ☐ Paediatrics ☐ Obs/ Gynae ☐ Surgery ☐ Infectious disease Clinician ☐

   Pathologist ☐ Others ☐ (Specify……………………………………..) Not applicable ☐
7. How long have practiced in your current profession:
8. What is your highest level of education? Diploma/HND ☐ Bachelor degree

   ☐ Masters/Membership ☐ PhD /Fellowship ☐ Others ☐ (Specify…………………………)
9. Have you ever received formal structured trained on antimicrobial stewardship or infectious disease management? Yes ☐ No ☐
10. In the last year, have you received any update training on antimicrobial use or stewardship practices?

    Yes ☐ No ☐

SECTION B: Knowledge of HCPs on Antimicrobial Stewardship Interventions

S/N	STATEMENT	Yes	No	Not sure	Correct Answers
1	Antimicrobial prescription audit with feedback is an antimicrobial stewardship program (ASP) intervention that can help persuade prescribers to prescribe antimicrobials rationally				Yes
2	Facility Antibiogram is useful to guide rational antibiotic use.				Yes
3	Antimicrobial automatic stop orders is a persuasive ASP intervention?				No
4	Prior authorization of restricted antimicrobials is an ASP strategy that can be used to reduce the rate of AMR development				Yes
5	Are you familiar with the WHO and/or Centers for Disease Control and Prevention (CDC) guideline for ASP?				Yes
6	Point prevalence survey of antimicrobials is a surveillance method that can be used in the hospital settings?				Yes
7	Do you now about the five antimicrobial prescribing decision for ensuring optimal antimicrobial use in a hospital setting?				Yes
8	Azithromycin is an antibiotic in the Access group of the WHO AWaRE classification of antibiotics?				No

9	De-escalation is not one of the ASP strategy of optimizing antimicrobial use to prevent AMR?				No
10	According to WHO classification of antimicrobials (AWaRE), nothing more 60% of antimicrobial consumption in a country must be from the Access Group.				No

SECTION C: Attitude of HCPs towards ASP assessment

S/N	STATEMENT	Strongly Agree	Agree	Neutral (Don't know)	Disagree	Strongly Disagree	Correct answers
1	Antimicrobial stewardship program (ASP) is a necessity in our hospital to ensure rational use of antimicrobials.						Strongly agree
2	ASP can help reduce the health care cost to patients.						Strongly agree
3	ASP can help reduce the adverse effects of inappropriate antimicrobial prescription.						Strongly agree
4	ASP threatens clinicians' autonomy.						Strongly disagree
5	Antimicrobial restrictions policies will negatively impact on patient care.						Strongly disagree
6	The antimicrobial treatment guidelines are difficult to implement in ideal healthcare setting.						Strongly disagree
7	I prefer to take my senior's advice rather than following antimicrobial guidelines.						Strongly disagree

SECTION D: Practices of HCPs of ASP assessment

S/N	STATEMENT	Yes	No	Correct Answers
1	Antimicrobials should routinely be prescribed when infections are suspected.			No
2	Rehydration and watchful waiting without empiric antibiotics is sufficient in most cases of watery diarrhea			Yes
3	Antibiotics should not be routinely prescribed for a patient with productive cough as it does not differentiate between bacterial and viral infection.			Yes
4	In an acute bronchitis assessment there is no need for chest X-ray, sputum culture, viral and serological analysis			No
5	Post-operative antimicrobial prophylaxis should be continued for more than 24 hours to effectively decrease the risk of surgical site infection.			No

6	Re-dosing of surgical antimicrobials should be considered during surgery if blood loss in patient >1.5L			Yes
7	I agree with the practice of the continuous use of antibiotic prophylaxis in a surgical patient as long as a wound drain tube is in situ for the purpose of preventing surgical site infection.			No
